# Complete chloroplast genome and phylogenetic analysis of *Acer leipoense* (Sapindaceae), a rare plant from Southwest China

**DOI:** 10.1080/23802359.2021.2017369

**Published:** 2022-01-05

**Authors:** Qiaoyun Liu, Ao Wang, Yongwei Gao, Liangcheng Zhao

**Affiliations:** a School of Ecology and Nature Conservation, Beijing Forestry University, Beijing, China; b Museum of Beijing Forestry University, Beijing Forestry University, Beijing, China

**Keywords:** *Acer leipoense*, chloroplast genome, endangered, phylogeny, Sapindaceae

## Abstract

*Acer leipoense* is a rare and endangered species of the Sapindaceae with a very restricted distribution in Sichuan, China. In this study, the complete chloroplast genome of *A. leipoense* was characterized by *de novo* assembly using high-throughput sequencing. The chloroplast genome was 155,702 bp in length; it contained a large single copy region (85,890 bp) and a small single copy region (18,100 bp), which were separated by a pair of 25,856-bp inverted repeat regions. A total of 128 genes were predicted, including 83 protein-coding genes, 37 tRNA genes, and eight rRNA genes. A phylogenetic analysis of 23 chloroplast genome sequences from the genus *Acer* revealed that *A. leipoense* was closely related to *A. yangbiense*.

*Acer* L. (family Sapindaceae) is one of the most diverse tree genera in the Northern Hemisphere; it contains more than 150 species, most of which are found in eastern Asia (Li et al. 2019). *Acer leipoense* Fang and Soong is an endemic species in southwestern China that has significant ornamental value. The natural distribution of *A. leipoense* is restricted to Leibo county, Ebian county and Tianquan county in Sichuan Province, China, where it is scattered in subtropical mixed forests at altitudes of 1900–2700 m (Fang 1966; Xu et al. 2008, and our investigations in 2018–2020). Due to over-harvesting and habitat degradation, *A. leipoense* is now rare and has been included in the *Threatened Species List of China’s Higher Plants* in the endangered (EN) category (Qin et al. 2017). To date, the chloroplast genome of *A. leipoense* has not been sequenced, and little is known about the phylogenetic relationships of this species. In this study, we assembled the complete chloroplast genome of *A. leipoense* using next-generation sequencing, providing a genetic resource for further genetic and conservation studies.

Fresh leaves of *A. leipoense* were collected from Erlang Mountian, Tianquan County, Sichuan province, China (29°59'19.6″ N, 102°38'34.1″E). The field study complied with national Wild Plant Protective Regulations and we were allowed by the Tianquan Forestry Bureau to collect the required samples of plant material. A specimen (voucher number: BJFUZLC055) was deposited at the Museum of Beijing Forestry University (Liangcheng Zhao, lczhao@bjfu.edu.cn). Total genomic DNA was extracted with a modified CTAB method (Doyle and Doyle 1987), and paired-end sequencing was performed on the Illumina HiSeq 2000 platform to obtain approximately 2.5 Gbp of high-quality *A. leipoense* reads. Genome assembly was performed using GetOrganelle v1.6.2.0 (Jin et al. 2020) with k-mer length 95, and all predicted genes were annotated using PGA (Plastid Genome Annotator) software (Qu et al. 2019) with the *Acer cesium* Wall. ex Brandis chloroplast genome (MN315269) as a reference. The complete annotated chloroplast genome of *A. leipoense* was deposited at GenBank under the accession number MZ725939.

The *Acer leipoense* chloroplast genome displays a typical quadripartite organization: one large single copy region (LSC) of 85,890 bp and one small single copy region (SSC) of 18,100 bp separated by two inverted repeats (IRs) of 25,856 bp each. The total length of the chloroplast genome is 155,702 bp; it encodes 128 genes, including 83 protein-coding genes, 37 transfer RNA (tRNA) genes, and eight ribosomal RNA (rRNA) genes. Among these genes, 15 (*atp*F, *ndh*A, *ndh*B, *pet*B, *pet*D, *rpl*16, *rpl*2, *rpo*C1, *rps*16, *trn*A-UGC, *trn*G-UCC, *trn*I-GAU, *trn*K-UUU, *trn*L-UAA, and *trn*V-UAC) contain one intron and three (*clp*P, *rps*12, and *ycf*3) have two introns. The total GC content of the chloroplast genome is 37.9%, and the GC contents of the LSC, SSC, and IR regions are 36.1%, 32.2%, and 43.0%, respectively.

To determine the phylogenetic position of *A. leipoense*, 23 complete chloroplast genome sequences (one newly generated and 22 obtained from GenBank) from *Acer* and two outgroup sequences from *Dipteronia* were used for phylogenomic analyses. The sequences were aligned with MAFFT (Katoh et al. 2019), and ambiguously aligned regions were trimmed with Gblocks v0.91b (Castresana 2000). Maximum likelihood (ML) trees were inferred with the best model (GTR + F + R4, identified with ModelFinder) using IQ-TREE (Nguyen et al. 2015), and branch support values were obtained from 1000 bootstrap replicates. Bayesian inference (BI) was performed with MrBayes v3.2.3 (Ronquist et al. 2012) using the GTR + G + I model; it was set to run for four million generations with four chains, sampled every 1000 generations, with 25% of the trees discarded as burn-in. Analysis was run to completion and the average standard deviation of the split frequencies was < 0.01. BI and ML analyses recovered the same tree topology (Figure 1). The results showed that *A. leipoense* was sister to *A. yangbiense* Chen and Yang with high support, consistent with morphological findings for the two species (Chen et al. 2003). *A. leipoense*, *A. yangbiense*, and *A. pilosum* Maxim. (1880) formed a strongly supported monophyletic group, which was located at the base of the clade formed by the other 20 *Acer* species.

**Figure 1.**
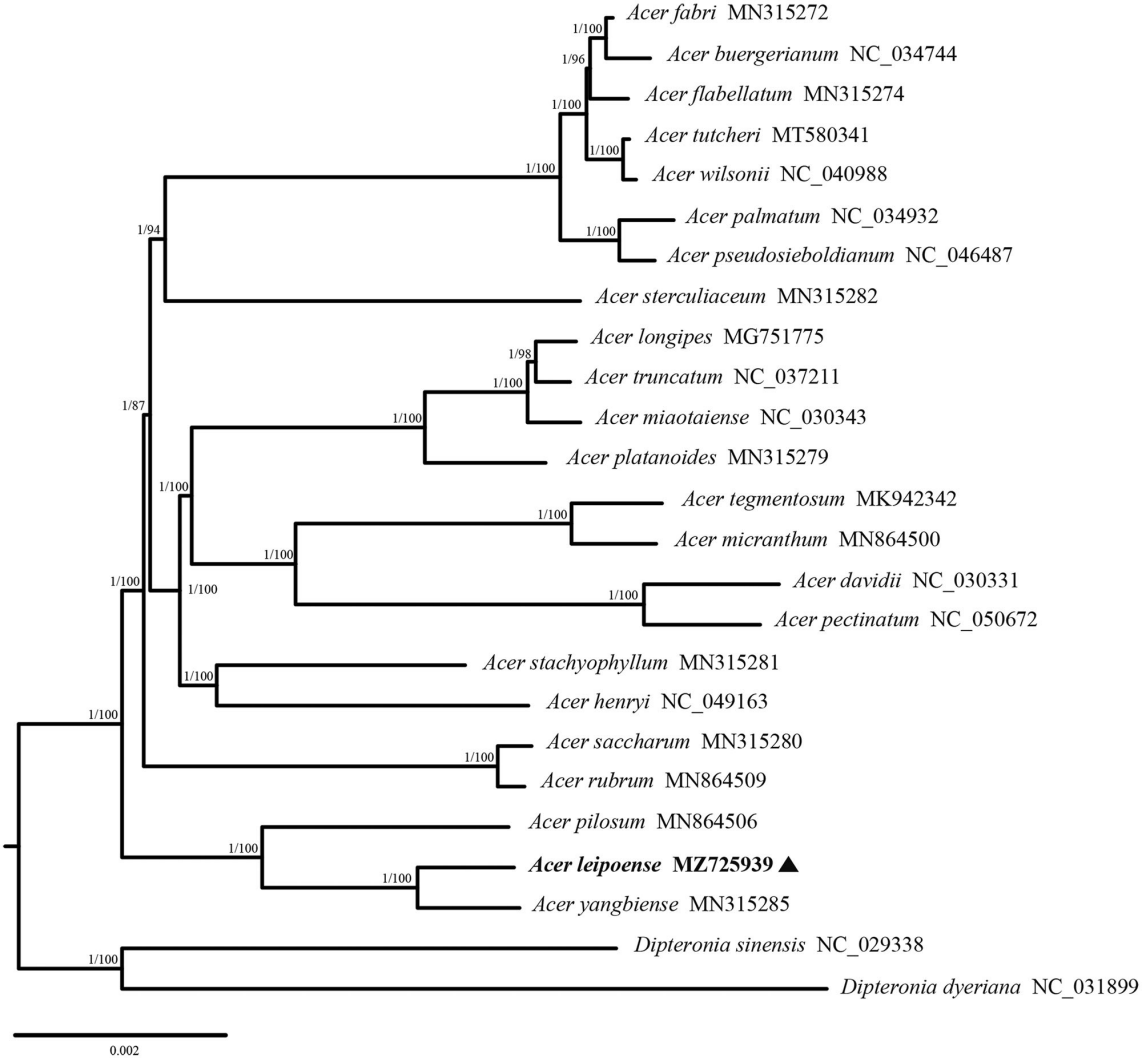
The maximum likelihood (ML) and Bayesian inference (BI) tree of 25 species inferred from the complete chloroplast genome sequences. Numbers associated with branches are bootstrap values and posterior probabilities.

## Data Availability

The genome sequence data that support the findings of this study are openly available in GenBank of NCBI at https://www.ncbi.nlm.nih.gov/ under the accession no. MZ725939. The associated BioProject, SRA, and Bio-Sample numbers are PRJNA752922, SRR15372360, and SAMN20667766, respectively.
